# Evidence against altered excitatory/inhibitory balance in the posteromedial cortex of young adult APOE E4 carriers: A resting state ^1^H-MRS study

**DOI:** 10.1016/j.ynirp.2021.100059

**Published:** 2021-12

**Authors:** A.G. Costigan, K. Umla-Runge, C.J. Evans, R. Raybould, K.S. Graham, A.D. Lawrence

**Affiliations:** a Cardiff University Brain Research Imaging Centre (CUBRIC), School of Psychology, Cardiff University, Maindy Road, Cardiff, CF24 4HQ, UK; b UK Dementia Research Institute, Cardiff, Hadyn Ellis Building, Maindy Road, Cardiff, CF24 4HQ, UK

**Keywords:** Alzheimer's disease, APOE E4, Default network, Glutamate, GABA, ^1^H-MRS

## Abstract

A strategy to gain insight into early changes that may predispose people to Alzheimer's disease (AD) is to study the brains of younger cognitively healthy people that are at increased genetic risk of AD. The Apolipoprotein (APOE) E4 allele is the strongest genetic risk factor for AD, and several neuroimaging studies comparing APOE E4 carriers with non-carriers at age ∼20–30 years have detected hyperactivity (or reduced deactivation) in posteromedial cortex (PMC), a key hub of the default network (DN), which has a high susceptibility to early amyloid deposition in AD. Transgenic mouse models suggest such early network activity alterations may result from altered excitatory/inhibitory (E/I) balance, but this is yet to be examined in humans. Here we test the hypothesis that PMC fMRI hyperactivity could be underpinned by altered levels of excitatory (glutamate) and/or inhibitory (GABA) neurotransmitters in this brain region. Forty-seven participants (20 APOE E4 carriers and 27 non-carriers) aged 18–25 years underwent resting-state proton magnetic resonance spectroscopy (^1^H-MRS), a non-invasive neuroimaging technique to measure glutamate and GABA *in vivo*. Metabolites were measured in a PMC voxel of interest and in a comparison voxel in the occipital cortex (OCC). There was no difference in either glutamate or GABA between the E4 carriers and non-carriers in either MRS voxel, or in the ratio of glutamate to GABA, a measure of E/I balance. Default Bayesian t-tests revealed evidence in support of this null finding. Our findings suggest that PMC hyperactivity in APOE E4 carriers is unlikely to be associated with, or possibly may precede, alterations in local resting-state PMC neurotransmitters, thus informing our understanding of the spatio-temporal sequence of early network alterations underlying APOE E4 related AD risk.

## Highlights

• Hyperactivity in posteromedial (PM) network linked to AD genetic risk (APOE E4).
• Such PM network hyperactivity may initiate pathogenic cascade that triggers AD.
• APOE mouse models suggest hyperactivity driven by excitatory/inhibitory imbalance.
• Using ^1^H-MRS at 3T we studied PM cortex E/I balance in young adult APOE E4 carriers.
• Found evidence against altered E/I balance in young adult APOE E4 carriers.

## 1 Introduction

The Apolipoprotein (APOE) E4 allele is the strongest genetic risk factor for late onset Alzheimer's disease (AD), where possession of one E4 allele increases risk of AD by ∼3–4 times, and two alleles by ∼12–14 times compared to the AD-neutral E3/E3 genotype (Belloy et al., 2019; Farrer et al., 1997). There is also a gene-dose effect on the age of onset of possible AD, where possession of 0, 1 and 2 E4 alleles reduces age of onset from 84 to 76 to 68 years, respectively (Corder et al., 1993; van der Lee et al., 2018). The mechanisms by which APOE increases AD risk are not clear. There is a growing consensus that AD-related brain changes and pathology occur decades before the onset of symptoms (Jack et al., 2018; Jagust, 2018; Masters et al., 2015; Sperling et al., 2014). By comparing APOE E4 carriers and non-carriers decades before the typical AD onset age, and presumably free from AD pathology, we can gain insight into differences that may predispose APOE E4 carriers to developing AD.

The posteromedial cortex (PMC) is a key region of interest for such studies in young people at increased AD genetic risk. The PMC (including retrosplenial cortex (RSC), posterior cingulate cortex (PCC) and precuneus (PCu)) (Parvizi et al., 2006) constitutes a major cortical hub that is densely connected with the medial temporal lobe to form a cortico-hippocampal brain network, referred to as the posterior medial (PM) network, a subsystem of the default network (DN) (Raichle, 2015; Ranganath and Ritchey, 2012). The PM network is critical to episodic memory and related cognitive processes relevant to AD (Ranganath and Ritchey, 2012; Ritchey and Cooper, 2020). The PMC is particularly susceptible to early amyloid (Aβ) plaque deposition, one of the hallmark pathologic features of AD (Buckner et al., 2005; Maass et al., 2019; Palmqvist et al., 2017), with APOE E4 allele carriers having both a younger age of onset and faster rates of PMC amyloid deposition relative to non-carriers (Burnham et al., 2020; Mishra et al., 2018). The cause of early Aβ aggregation within PMC is currently unknown, but may reflect a lifespan regional vulnerability (Buckner et al., 2005; Sepulcre et al., 2018).

Aberrant PMC network activity is seen consistently in individuals at high risk for AD and during the early stages of the disease. Functional magnetic resonance imaging (fMRI) studies reveal reduced PMC deactivation in conjunction with hippocampal hyperactivation during episode encoding tasks (see Palop and Mucke, 2016; Zott et al., 2018 for review). Inadequate deactivation of the PMC is associated with amyloid deposition and poorer episodic memory performance, and has been related to conversion from mild cognitive impairment (MCI) to AD (Palop and Mucke, 2016; Foster et al., 2017; Zott et al., 2018). In later stages of AD, the reduced DMN deactivation may persist, whereas the hippocampus is hypoactive during memory encoding (Palop and Mucke, 2016; Zott et al., 2018).

Such PMC functional alterations, mirroring the functional signature of early AD, may begin decades before the clinical onset of AD in participants at increased AD risk. For example, several fMRI studies have found a lack of PMC/DN deactivation and hippocampal hyperactivity during episodic encoding and spatial memory tasks in young adult (aged 20–30 years) APOE E4 carriers relative to non-carriers (Filippini et al., 2009; Kunz et al., 2015; Persson et al., 2008; Shine et al., 2015; see McDonough et al., 2020 for review), alongside increased PMC network connectivity in APOE E4 carriers during the resting state (Filippini et al., 2009; Zheng et al., 2021) (but see Mentink et al., 2021 for a failure to replicate and Kucikova et al., 2021 for wider review). The pattern of reduced PMC/DN deactivation has also been detected in participants with familial AD (FAD) mutations (Presenilin1, PSEN1) decades before the typical age-of-onset of AD (Mondadori et al., 2006; Reiman et al., 2012). As the functional alterations in the PM network in people at risk of developing AD widely overlap with regions that ultimately develop AD pathology and hypometabolism/atrophy, this hyperactivity may be a harbinger and even a cause of AD rather than a compensatory phenomenon (Buckner et al., 2005; Jagust and Mormino, 2011; Palop and Mucke, 2016).

Indeed, AD mouse models provide evidence that hyperactivity is causally linked to/drives amyloid deposition. In APP transgenic mice expressing a mutated form of Aβ precursor protein, lactate (as a measure of neuronal activity) was closely associated with interstitial fluid (ISF) Aβ levels, and in turn, ISF Aβ predicted region-specific Aβ deposition, particularly in PMC (Bero et al., 2011). In addition, chronic optogenetic activation in young FAD mice promotes amyloid deposition (Yamamoto et al., 2015). A recent study of MCI patients provided human evidence that hyperactivity is associated with subsequent amyloid deposition (Leal et al., 2017). Collectively, these studies support the hypothesis that Aβ accumulates in a concentration-dependent manner throughout life, with selective neuronal and network hyperactivity in vulnerable individuals leading to excessive Aβ production that eventually precipitates a deleterious cascade involving tau pathology and neurodegeneration (Jagust and Mormino, 2011; Selkoe and Hardy, 2016; Busche and Hyman, 2020).

It is important, therefore, to better understand the causes of the PMC hyperactivity detected in young APOE E4 carriers, as this could provide important insight into the earliest changes that may predispose to AD. One such mechanism that may underpin PMC hyperactivity is alterations in the levels of the inhibitory neurotransmitter, γ-aminobutyric acid (GABA), and excitatory neurotransmitter, glutamate. The balance between these neurotransmitters, termed the excitatory/inhibitory (E/I) balance, mediates normal neural network activity (Busche and Konnerth, 2016; Palop and Mucke, 2016). A growing consensus views AD as a circuit-based disorder, where an alteration of the physiological E/I balance underlies both the functional impairment of local neuronal circuits as well as that of large-scale networks in the amyloid-depositing brain (Busche and Konnerth, 2016; Harris et al., 2020; Palop and Mucke, 2016; Lauterborn et al., 2021). Mouse models of AD suggest that a shift in E/I balance towards excitation initially causes hyperactivity in cortical and hippocampal neurons, prior to the appearance of amyloid plaques (with hypoexcitability in late disease stages, linked to subsequent tau deposition) (Bi et al., 2020; Busche and Konnerth, 2016; Harris et al., 2020; Jiménez-Balado and Eich, 2021; Najm et al., 2019; Palop and Mucke, 2016).

The APOE E4 genotype may be a distinct risk factor for hyperexcitability (Toniolo et al., 2020), linked both to an increase in excitatory tone and a decrease in GABAergic inhibition. For example, a study combining fMRI with electrophysiology identified that entorhinal cortex hyperactivity in APOE E4 mice, in the absence of overt AD pathology, was associated with decreased response to inhibitory GABAergic inputs on pyramidal neurons, rather than increased excitability due to differences in NMDA glutamate receptors (Nuriel et al., 2017). Alternatively, impairment in glutamate production in APOE E4 transgenic mice has been suggested, as E4 compared to E3 mice had a lower concentration of glutamate and glutaminase, the enzyme that converts glutamine to glutamate, and a higher concentration of glutamine (Dumanis et al., 2014). Thus, the levels of local neurotransmitters in young APOE E4 carriers could provide important insight into why the PMC demonstrates hyperactivity, and why this is a key region affected early by amyloid pathology (Burnham et al., 2020).

In humans, the concentration of neurotransmitters can be measured non-invasively *in vivo* using proton magnetic resonance spectroscopy (^1^H-MRS) (Stagg and Rothman, 2014). Glutamate is quantified as “Glx”- a composite measure of glutamate plus glutamine (due to their largely overlapping signals in the MRS spectrum making them difficult to quantify separately) (Rae, 2014; Stagg and Rothman, 2014), and GABA as “GABA+", which consists of GABA plus co-edited macromolecules (Mullins et al., 2014). Glx and GABA+ quantified via ^1^H-MRS in the PMC in the resting state have been associated with both the magnitude of the local BOLD response during task-related fMRI and network-level resting functional connectivity, showing that regional excitation-inhibition balance predicts default network deactivation and intrinsic connectivity. In these PMC studies, higher concentrations of inhibitory GABA+ were associated with greater task-related deactivation, higher levels of excitatory Glx with less deactivation, and a higher E/I (i.e. Glx/GABA+) ratio with less deactivation (Gu et al., 2019; Hu et al., 2013). Local PMC GABA and Glx concentration also predicted network-level resting functional connectivity, with Glx correlating positively and GABA+ correlating negatively with PMC resting functional connectivity (Kapogiannis et al., 2013).

An unexplored avenue is whether the PMC task-related hyperactivity/reduced deactivation and resting hyperconnectivity seen in young APOE E4 carriers could reflect altered GABA+ and or Glx levels and a shift in E/I balance toward excitation, as suggested by animal models of AD genetic risk.

^1^H-MRS studies of E/I neurotransmission in older individuals with APOE E4, MCI and AD have produced inconsistent results, and are confounded by the presence of AD pathology and other age-related brain changes. Several studies show that AD and MCI individuals have lower PMC GABA+ and Glx than age-matched healthy controls (Bai et al., 2014; Londono et al., 2013; Oeltzschner et al., 2019; Riese et al., 2015). It is less clear whether older APOE E4 carriers have altered PMC GABA+ and Glx levels prior to amyloid pathology: E4 carriers appeared to have lower PMC GABA+ and Glx compared to non-carriers at ∼age 70, however this was based on a small sample size of nine E4 carriers and was not statistically significant (Riese et al., 2015). PMC Glx was compared in a larger sample of E4 carriers and non-carriers at ages 20–70 (split into young and old groups). No difference in PMC Glx was found between APOE groups when collapsed across age groups, and there was no significant interaction between APOE genotype and age on PMC Glx (despite a main effect of age on Glx whereby older participants had lower PMC Glx) (Suri et al., 2017). A published abstract found that higher levels of GABA and Glu were associated with higher amyloid burden in a group of 30 older individuals, which was particularly evident in APOE4 carriers, but this study did not examine Glu/GABA (E/I) ratio (Schreiner et al., 2016). Two previous studies have investigated other ^1^H-MRS metabolites in the PMC in young APOE E4 carriers (Calderon-Garciduenas et al., 2015; Suri et al., 2017; see Supplementary Material, Section 6 for replication), however this study is the first to measure both Glx and GABA+, and the E/I balance (Glx/GABA+ ratio) in the PMC using ^1^H-MRS.

Based on several findings of reduced PMC deactivation in young APOE E4 carriers, and evidence suggesting this may be linked to an E/I imbalance, we hypothesized that young APOE E4 carriers would show a shift in E/I balance toward excitation due to either/both lower PMC GABA+ and higher Glx than non-carriers. This would be reflected by an increased PMC E/I (Glx/GABA+) ratio in E4 carriers compared to non-carriers. To assess regional specificity of any difference, as recommended in Duncan et al. (2014), we used a comparison voxel, placed outside the PMC in the occipital cortex (OCC). The OCC is a region not reported to show altered BOLD in fMRI studies of young APOE E4 carriers relative to non-carriers (Filippini et al., 2009; Kunz et al., 2015; Shine et al., 2015).

## 2 Materials and methods

### 2.1 Participants

Participants were recruited from two cohorts of undergraduate Psychology students who provided a saliva sample for APOE-genotyping. The first cohort (total n=125) was the same as that in Shine et al. (2015), and Hodgetts et al. (2019), and the second cohort comprised a further new 229 students. Nineteen participants (10 APOE E4 carriers and 9 non-carriers) from the first cohort and 28 (10 APOE E4 carriers and 18 non-carriers) from the second cohort took part in this study. The total sample size, therefore, was 20 APOE E4 carriers and 27 non-carriers (see Table 1). There was one male in each group, reflecting the predominantly female undergraduate Psychology student cohort.

**Table 1:** Demographic details of the APOE E4 carrier and non-carrier groups, and number of participants scanned in each group. Statistical comparison between groups show no significant difference in age or gender between APOE E4 carrier and non-carrier groups. BF = Bayes Factor.

	APOE E4 carriers	Non-carriers	Statistics
Total n	20	27	
Genotype split	1 E4/E4, 18 E3/E4, 1 E2/E4	24 E3/E3, 3 E2/E3	
N females/males	19/1	26/1	X^2^(1, N=47)=0.047, p=0.83, BF_01_=4.25
Age (years; mean ± SD)	21.2 ± 1.9	19.9 ± 1.6	t(45)=1.14, p=0.26, BF_01_=2.00

APOE E4 carriers and non-carriers were matched for age, family history of dementia, and family history of psychiatric illness. Participants were excluded if they had a self-reported history of depression or psychiatric illness or were taking any psychoactive medication. All participants were right-handed, with normal or corrected-to-normal vision.

A double-blind strategy was adopted for this study, whereby both participants and researchers collecting and analysing data were blind to the participants’ APOE-genotypes, in order to prevent any bias during analyses. The study received ethical approval from the Cardiff University School of Psychology Research Ethics Committee, and all participants provided written informed consent.

The sample size was estimated via a priori power calculation, using G*Power version 3.1.9.7 (Faul et al., 2009). Based on the fMRI study of Shine et al. (2015) in a similarly aged population, which detected a very large effect size (Cohen's d > 1) for the difference in PMC deactivation between APOE E4 carriers and non-carriers, we planned our experiment to detect a conventionally large effect (Cohen's d = 0.8) while safeguarding against effect size inflation in published studies (Perugini et al., 2014, 2018). We initially aimed for a sample size of n = 26 per group, which would have 80% power to detect such a large effect at p < 0.05 using a 2-tailed *t*-test. The final obtained sample of 20 APOE E4 carriers and 27 non-carriers was similar to previous ^1^H-MRS studies in young APOE E4 carriers measuring other metabolites (tNAA, mI, Cho, tCr), in which the sample sizes were 8 APOE E4 carriers vs 22 non-carriers (Suri et al., 2017), and 22 APOE E4 carriers vs 28 non-carriers (Calderon-Garciduenas et al., 2015), as well as a study of GABA and glutamate in older individuals without cognitive impairment (Quevenco et al., 2019).

### 2.2 APOE genotyping

Procedures for DNA extraction from saliva and APOE genotyping were the same for the two cohorts. DNA was obtained from saliva using Oragene OG-500 saliva kits (DNA Genotek, Inc., Ontario, Canada). DNA extraction and APOE-genotyping were performed in the MRC Centre for Neuropsychiatric Genetics and Genomics at Cardiff University. Since APOE isoforms differ due to a single nucleotide polymorphism (SNP) at two sites in the gene, a single SNP genotyping assay was performed for each site to determine APOE genotype. The SNP rs429358 was determined by KASP genotyping and rs7412 by Taqman genotyping. These were detected on Tecan infinite F200 pro and StepOnePlus™ Real-Time PCR System platforms, respectively. Haplotypes corresponding to APOE E2, E3 and E4 were then deduced.

Genotyping was successful in 100/125 and 224/229 participants from the two cohorts respectively. The distribution of genotypes of those successfully genotyped in the first cohort was E2/E2 (1/100, 1%), E2/E3 (10/100, 10%), E2/E4 (1/100, 1%), E3/E3 (69/100, 69%), E3/E4 (19/100, 19%), and E4/E4 (0/100, 0%). The genotype-distribution in the second cohort was E2/E2 (0/224, 0%), E2/E3 (38/224, 17%), E2/E4 (7/224, 3%), E3/E3 (125/224, 56%), E3/E4 (52/224, 23%), and E4/E4 (2/224, 1%).

### 2.3 MRI scan acquisition

All scans were performed at the Cardiff University Brain Research Imaging Centre (CUBRIC) on a 3T General Electric (GE) HDx scanner fitted with an 8-channel phased array head coil. A high resolution anatomical MRI scan was obtained for each participant using a 3D T1-weighted (T1w), fast spoiled gradient echo (FSPGR) sequence (TE/TR = 3.0/7.9 ms; TI=450 ms; flip angle 20°; data matrix 256x192x176; field of view 256x192x176mm^3^; acquisition time approx. 7 min). The FSPGR was used to aid ^1^H-MRS voxel placement during scanning (see Section 2.5).

The phase of menstrual cycle has been suggested to have an impact on ^1^H-MRS metabolite concentrations (Batra et al., 2008; De Bondt et al., 2015; Epperson et al., 2002), therefore scans of female participants were scheduled during their luteal phase (days 15–28 of the cycle, where day 1 was defined as the first day of menstruation). No restrictions were placed on scan scheduling if female participants were taking the contraceptive pill, as GABA+ concentration does not significantly differ between pill-on and pill-free days (De Bondt et al., 2015).

### 2.4 ^1^H-MRS acquisition

**Fig. 1:**
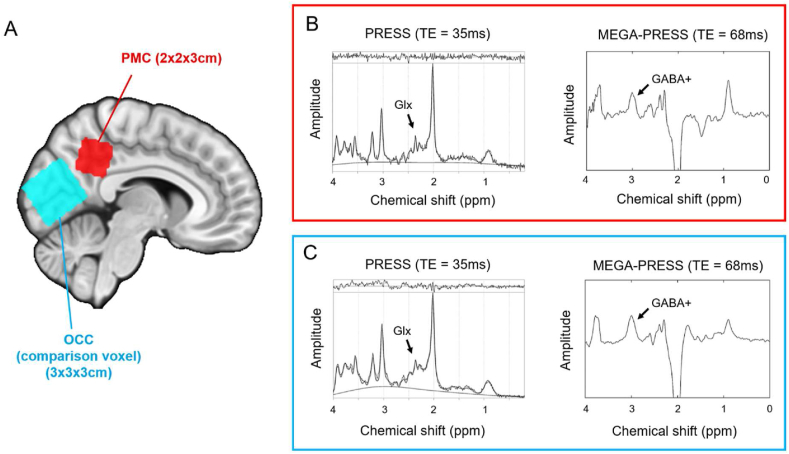
^**1**^**H-MRS voxel placement and**^**1**^**H-MRS spectra.** (A) ^1^H-MRS voxel placement for one representative participant. Voxels have been transformed into MNI standard space and overlaid on the MNI152 2 mm standard brain template (Grabner et al., 2006). Example of a PRESS and MEGA-PRESS spectrum from (B) the PMC voxel and (C) OCC voxel in one participant.

Single voxel proton spectra were acquired from the PMC (the voxel of interest, measuring 2x2x3cm^3^), and the occipital cortex (OCC, the comparison voxel, measuring 3x3x3cm^3^) in the resting-state. Examples of voxel placement are shown in Fig. 1.

Landmarks used for voxel placement were consistent with Costigan et al. (2019), which was an approach developed from a pilot study assessing test-retest reliability of voxel placement and metabolite concentrations (see Supplementary Material, Section 5). Briefly, an odd number of AC-PC aligned slices were acquired from the bottom to the top of the corpus callosum (typically 5 or 7 slices). The PMC voxel was placed on the plane of the middle slice, and adjusted to lie posterior to the ventricles, ensuring it was not covering an area of CSF to prevent artefacts in the ^1^H-MRS spectrum. The OCC voxel was placed above the line of the tentorium cerebelli and adjusted so it did not contain any scalp tissue, which would have resulted in lipid contamination in the spectra.

In each voxel, one point-resolved spectroscopy (PRESS) scan was obtained to measure Glx (the combined signal of glutamate + glutamine) (TE/TR = 35/1500 ms; number of averages = 128; scan time 4mins) (Bottomley, 1984). One Mescher-Garwood PRESS (MEGA-PRESS) scan (Mescher et al., 1998; Rothman et al., 1993) was obtained to measure GABA + coedited macromolecules, “GABA+", (TE/TR = 68/1800 ms; OCC 166 edit on/off pairs, scan time 10mins; PMC 256 on/off pairs, scan time 15mins). Shimming was performed before all ^1^H-MRS scans to ensure water-linewidth of 10Hz or lower, in order to obtain sharp peaks in the resulting ^1^H-MRS spectrum.

Different MRS sequences were used to quantify Glx and GABA+ so that the most optimal method was used for each metabolite. The MEGA-PRESS spectral editing acquisition was necessary because GABA+ is challenging to quantify accurately using standard PRESS methods, due to its very low concentration, and its peak resonances in the MRS spectrum have a low amplitude and overlap with other metabolites (mainly by creatine at 3.0ppm) (Harris et al., 2017; Mullins et al., 2014). MEGA-PRESS acquisitions include additional editing pulses placed symmetrically about the water resonance (4.7ppm) resulting in editing pulses at 1.9ppm (edit on) and at 7.5ppm (edit off) in order to subtract the creatine peak, enabling accurate GABA+ detection and quantification.

A separate PRESS sequence was used to quantify Glx, rather than using the MEGA-PRESS edit off scan as used in some studies, because short TE PRESS scans (e.g. TE 35 ms, rather than TE 68 ms as in the MEGA-PRESS edit off scan) have been found to be more accurate for our voxel of interest. For example, a test-retest reliability study found the 35 ms PRESS scan produced a lower coefficient of variation and lower Cramer-Rao Lower Bounds (CRLBs) for PMC Glx over three intra-scan repeats than PRESS scans with a longer TE (Hancu, 2009).

The difference in voxel sizes was a trade-off between voxels being large enough to have a good signal-to-noise ratio (SNR) for accurate metabolite quantification, yet not too large, to maintain spatial specificity. The 3x3x3cm OCC MEGA-PRESS protocol has reliably produced good quality spectra to quantify GABA+ (e.g. Muthukumaraswamy et al., 2009; Muthukumaraswamy et al., 2012) and has been recommended for MEGA-PRESS scanning (Mullins et al., 2014). A PMC voxel of this size, however, would have reduced the spatial specificity of our region of interest, and overlapped with the OCC voxel. As the spatial specificity of the PMC voxel was of key importance here, its size was reduced to 2x2x3cm, which is more consistent with the PMC voxel size employed in previous studies of APOE carriers, and MCI or AD patients (Kantarci et al., 2000, 2002; Suri et al., 2017; Voevodskaya et al., 2016). To counteract the reduction in SNR from this volume reduction, the length of the PMC MEGA-PRESS scan was increased to make the SNR similar across the two MRS voxels.

### 2.5 Data analysis

PRESS data were analysed using TARQUIN (Totally Automatic Robust Quantification In NMR) version 4.3.3 (Wilson et al., 2011). Glx data were excluded if the CRLB was above 20%, consistent with the data quality criteria commonly found in the ^1^H-MRS literature (Cavassila et al., 2001; Lin et al., 2021; Near et al., 2020).

MEGA-PRESS data were analysed using GANNET (GABA-MRS Analysis Tool) version 2.0 (Edden et al., 2014). Data quality was assessed by two independent raters (authors AGC and CJE) using a 3-point rating scale (very good, satisfactory, reject; as in Lipp et al., 2015). Inter-rater reliability was assessed via the coefficient of variation (CV), which found good correspondence between raters for both PMC (CV=8.46%) and OCC (8.27%).

Metabolite concentrations were corrected for voxel composition: using each participant's high resolution T1w anatomical MRI scan, FSL's FAST tool segmented the areas of the PMC and OCC ^1^H-MRS voxels into cerebrospinal fluid (CSF), grey matter (GM) and white matter (WM) (Zhang et al., 2001). Metabolites were quantified using the tissue H_2_O signal as an internal concentration reference and are expressed as a concentration in millimoles (mM) per unit tissue volume (Near et al., 2020). The water signal was preferable here, as opposed to using tCr as the reference metabolite, to prevent ambiguity for whether there were alterations in the numerator (i.e. Glx or GABA+) or denominator (tCr) between APOE carriers and non-carriers (Wilson et al., 2019). Furthermore the water signal has a much higher signal than that of the tCr peak, therefore is more reliable for quantification (Near et al., 2020). The metabolite signals were corrected for the proportion of CSF in the voxel (as the concentration of metabolites in CSF is negligible) and the water reference signal was corrected to account for the differing water content of CSF, GM and WM (Near et al., 2020; Wilson et al., 2019).

### 2.6 Statistics

The metabolites Glx, GABA+ and the ratio of Glx/GABA+ (as a measure of E/I balance (Steel et al., 2020)) were compared between APOE E4 carriers and non-carriers in each voxel using Student's two-tailed independent sample t-tests in SPSS version 26. Cohen's d was calculated as a measure of effect size. To support the frequentist statistics, Bayesian t-tests were implemented in JASP version 0.13.1. The Bayes factor (BF) assesses the strength of the evidence that the data provide for the null hypothesis (H0, i.e. no difference between APOE groups) versus the alternative hypothesis (H1, i.e. difference between groups), expressed as BF_01_, or for the alternative versus the null hypothesis, denoted BF_10_. Where the frequentist *t*-test was non-significant, BF_01_ is reported to support this null finding. Bayes factors grade the strength of evidence on a continuous scale (with a BF_01_ of 1 indicating the finding is equally likely under H0 and H1), although a BF_01_ value over 3 is frequently interpreted as substantive evidence for the null hypothesis (Hu et al., 2018; Keysers et al., 2020). Graphs were created using GraphPad Prism version 5.01 for Windows, GraphPad Software, San Diego California USA, www.graphpad.com.

## 3 Results

### 3.1 Participants and data quality exclusions

The genotype split of the 20 APOE E4 carriers and 27 non-carriers is shown in Table 1. There was no significant difference in age between groups, nor in the proportion of males in each group (see Table 1).

Data quality assessments resulted in the exclusion of three PMC Glx (one E4 carrier) and two OCC Glx (two E4 carriers) scans, and 14 PMC GABA+ (six E4 carriers) and one OCC GABA+ (E4 carrier) scans. One PMC GABA+ scan was excluded as an outlier (GABA+ concentration was 4 standard deviations from the mean). Final sample sizes for each metabolite in the two MRS voxels are shown in Table 2.

**Table 2:** **Metabolite results, sample sizes and summary of statistical analysis.** Metabolite values are shown as the mean concentration (mM) and standard error of the mean. Sample sizes indicate the number of good quality data sets remaining after data quality assessment and outlier exclusion. Statistical tests are a 2-tailed *t*-test and a 2-tailed Bayesian *t*-test to assess the strength of the evidence for the null hypothesis (i.e. no difference between groups, denoted BF_01_).

MRS voxel	PMC				OCC			
	E4 carriers	Non-carriers	*t*-test p value	BF_01_ (evidence for null)	E4 carriers	Non-carriers	*t*-test p value	BF_01_ (evidence for null)
**Glx**	20.86	21.21	0.75	3.22	21.01	20.59	0.75	3.24
St err	0.82	0.74			0.92	0.87		
n	19	26			19	27		

**GABA+**	2.07	2.03	0.83	2.90	1.91	1.96	0.38	2.47
St err	0.13	0.11			0.03	0.05		
n	14	18			19	27		

**Glx/GABA+**	10.67	10.61	0.96	2.93	11.08	10.62	0.54	2.89
St err	0.86	0.59			0.52	0.50		
n	14	17			19	27		

### 3.2 Regional specificity of PMC and OCC metabolites

There were no significant correlations between PMC metabolites and OCC metabolites, suggesting regional specificity of metabolite concentrations: PMC vs OCC Glx, r(45)=0.22, p=0.15, BF_01_=1.97; PMC vs OCC GABA+, r(33)=0.13, p=0.47, BF_01_=3.53; PMC vs OCC Glx/GABA+, r(33)=0.24, p=0.19, BF_01_=2.00.

### 3.3 Glx

**Fig. 2:**
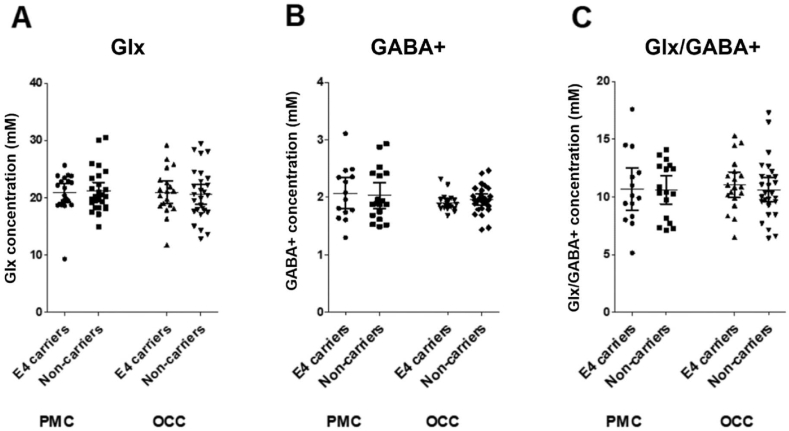
**Comparison of metabolite concentrations in APOE-E4 carriers and non-carriers in the voxel of interest, the PMC, and the comparison voxel in the OCC.** (A) Glutamate (Glx); (B) GABA+; (C) the ratio of Glx to GABA+ as a measure of excitatory/inhibitory (E/I) balance. Each dot represents one participant, the horizontal lines represent the mean and the error bars are 95% confidence intervals.

There was no statistically significant difference in PMC Glx between APOE E4 carriers and non-carriers: t(43)=0.32, p=0.75; Cohen's d= −0.10; BF_01_ = 3.22. There was also no statistically significant difference in Glx between APOE groups in the OCC comparison voxel: t(44)=0.32, p=0.75; Cohen's d=0.10, BF_01_ = 3.24 (see Fig. 2A).

### 3.4 GABA+

There was no statistically significant difference in PMC GABA+ between E4 carriers and non-carriers: t(30)=0.22, p=0.83; Cohen's d=0.08, BF_01_ = 2.90. This was also the case for GABA+ in the OCC comparison voxel: t(44)=0.88, p=0.38, Cohen's d= −0.26, BF_01_ = 2.47 (See Fig. 2B).

### 3.5 Excitatory/inhibitory (E/I) balance (Glx/GABA+)

There was no statistically significant difference in the ratio of PMC Glx/GABA+ between E4 carriers and non-carriers: t(29)=0.05, p=0.96, Cohen's d=0.02, BF_01_=2.93. Similarly, there was no statistically significant difference in Glx/GABA+ in the OCC comparison voxel: t(44)=0.62, p=0.54, Cohen's d=0.19, BF_01_ = 2.89 (See Fig. 2C).

## 4 Discussion

Using ^1^H-MRS we tested the hypothesis that there would be PMC GABA+ and/or Glx and consequent Glx/GABA+ ratio differences between young adult APOE E4 carriers and non-carriers measured in the resting state. Specifically, we predicted APOE E4 carriers would show lower PMC GABA+ and/or higher Glx and Glx/GABA+ ratio than non-carriers, resulting in altered E/I balance favouring excitation. This was based on converging lines of evidence: previous fMRI studies of young adult APOE E4 carriers showing PMC hyperactivation/reduced deactivation (Filippini et al., 2009; Persson et al., 2008; Shine et al., 2015; but see Mentink et al., 2021); fMRI-^1^H-MRS findings that levels of task-related PMC deactivation are related to local resting Glx and GABA+ concentrations and resultant E/I balance (Gu et al., 2019; Hu et al., 2013); and circuit-based models of AD, based on studies of transgenic AD mice, which suggest that early hyperactivity is linked to alterations in local neurotransmitters, causing physiological E/I imbalance and network hyperexcitability (Andrews-Zwilling et al., 2010; Busche and Konnerth, 2016; Li et al., 2009; Nuriel et al., 2017; Palop and Mucke, 2016).

Counter to our predictions, our results provide evidence to support the null hypothesis of no difference in PMC (or OCC) neurotransmitter concentrations between APOE E4 carriers and non-carriers. This was found using frequentist statistics (p > 0.7 in PMC), supported by very low effect sizes (Cohen's d<0.1 in PMC) and importantly by Bayes factors in favour of the null of ∼ 3 (BF_01_ range from 2.9 to 3.2 in PMC). The Bayes factor (BF_01_) provides a continuous measure of evidence for H0 over H1 (and vice versa) (Dienes, 2014; Dienes and Mclatchie, 2018). While there are no necessary thresholds (in contrast to the fixed significance levels of the frequentist approach) to interpret BFs, several authors have suggested that a BF_01_ > 3 is “substantial” evidence in favour of the null (Dienes and Mclatchie, 2018). Therefore, despite the relatively modest sample size, our findings have considerable evidential value (Dienes, 2014; Dienes and Mclatchie, 2018).

There are several potential explanations for our null results, some of which may reflect limitations of ^1^H-MRS studies in general, while some of which may have important implications for the spatial and temporal evolution of AD-related biomarkers across the lifespan in APOE E4 carriers (Harris et al. 2020; Jack et al., 2013; Jagust and Mormino, 2011).

Turning first to the implications of our study for APOE and AD, our finding of no difference between PMC Glx, GABA+ and E/I balance in APOE groups could inform the timeline of spatiotemporal evolution of hyperactivity differences in APOE E4 carriers. We predicted that alterations in E/I balance would account for the previously established alterations in PMC deactivation seen in young adult E4 carriers (e.g. Shine et al., 2015), based on transgenic APOE mouse work showing, for example, that APOE E4 contributes to neuronal hyperactivity by diminishing inhibitory tone, even independently of amyloid (and tau) pathology (Bi et al., 2020; Hijazi et al., 2020; Jiménez-Balado and Eich, 2021; Nuriel et al., 2017). However, detecting no difference in PMC neurotransmitters in young APOE E4 carriers indicates that task-related PM network hyperactivity may not straightforwardly reflect a difference in underlying Glx and/or GABA+ levels. For example, Najm et al. (2019) propose that tau pathology (which may be present even in young adulthood - Braak and Del Tredici, 2011) and mitochondrial impairment occur prior to GABA-ergic cell loss, which then results in E/I imbalance and network hyperexcitability. Other work suggests a complex and potentially synergistic relationship between accumulation of soluble amyloid oligomers, tau and hyperactivity (Busche and Konnerth, 2015; Harris et al., 2020; Palop and Mucke, 2016; Zott et al., 2018). Thus perhaps early subtle AD-related pathology leads to reduced PMC deactivation via fMRI and contributes to a shift in E/I balance, which further contributes to a complex bidirectional relationship between hyperactivity and pathology that only subsequently is detectable via ^1^H MRS later in the lifespan (Quevenco et al., 2019; Schreiner et al., 2016).

Relatedly, APOE E4 may also impact PMC function independent of local neurotransmitter levels and E/I balance. Such factors may include cerebrovascular dynamics (e.g. cerebral blood flow and volume (CBF and CBV), cerebrovascular reactivity (CVR)), energy consumption by neurons and glia, and neuronal firing dynamics (rate, amplitude, frequency, phase) (Ekstrom, 2021; Singh, 2012; Stiernman et al., 2021)). PMC CVR could be a good avenue for further investigation, given alterations in older APOE E4 carriers and increased permeability of the blood-brain-barrier in AD and APOE E4 carriers (Korte et al., 2020; Montagne et al., 2020; Tai et al., 2016; Thambisetty et al., 2010). Two previous studies on the vasculature have detected that young APOE E4 carriers have lower whole brain grey matter CBF (Chandler et al., 2019), and lower hippocampal CO2-CVR in a memory-encoding task during a CO2 challenge (Suri et al., 2015), but the potential impact of such changes on PMC deactivation has yet to be investigated.

Another potential explanation for our null finding is that the PMC hyperactivation (reduced deactivation) seen in young APOE E4 carriers reflects not local cortical E/I imbalance, but a *downstream* consequence of initial hyperactivity elsewhere, specifically the medial temporal lobe (MTL). There is dense reciprocal structural and functional connectivity between the MTL and PMC (Bubb et al., 2017; Parvizi et al., 2006; Wang et al., 2016) and network interactions between these regions are important for episodic memory (Ritchey and Cooper, 2020). Network-based accounts of AD suggest that hippocampal hyperexcitability can impact connected PMC regions (e.g. Pasquini et al., 2019) and that AD pathology may spread from the MTL to posterior DMN regions via PM network connectivity (Braak & Braak, 1991, 1995; Harris et al., 2010; Seeley, 2017). Given evidence of hippocampus and entorhinal cortex hyperactivity on fMRI in young APOE E4 carriers (Filippini et al., 2009; Kunz et al., 2015), as well as increased resting parieto-temporal connectivity on magnetoencephalography (MEG) (Koelewijn et al., 2019), alongside preclinical evidence that young adult transgenic AD rodents show hippocampal hyperexcitability (Haberman et al., 2017) and that APOE E4 mice show prominent early entorhinal cortex hyperactivity linked to reduced inhibitory tone (Nuriel et al., 2017), it would be interesting to study the effect of hippocampal E/I balance on both hippocampal BOLD and its distal effect on PMC BOLD in young APOE E4 carriers. Some evidence suggests that resting hippocampal Glx levels can influence patterns of cortico-hippocampal connectivity on fMRI (Nikolova et al., 2017; Wagner et al., 2016) but the influence of APOE E4 is not yet known. The hippocampus is, however, a challenging brain region to study via ^1^H-MRS, as it suffers from lower SNR due to smaller voxel size required for spatial specificity, and large susceptibility effects leading to broader linewidths and lower spectral resolution (Bednařík et al., 2015). Future ^1^H-MRS studies in the hippocampus and related regions could benefit from higher magnetic field strength (e.g. 7T) to improve SNR and spectral quality.

Turning next to ^1^H-MRS limitations, our null finding could in part be due to ^1^H-MRS at 3T not being sufficiently sensitive or specific enough to detect true PMC GABA+ or Glx difference between APOE groups. ^1^H-MRS quantified Glx and GABA+ indicate the metabolite concentrations within the MRS voxel, whereas other aspects of neurotransmission, including glutamate or GABA receptor density at the synapse or binding to receptors, are not accounted for. Such processes cannot be measured using MRI, but could be examined using molecular imaging with PET (e.g. Cuypers et al., 2021). In addition, the Glx and GABA+ measured via ^1^H-MRS indicates MRS-visible pools, which may not all be involved in neurotransmission, as these may include Glx and GABA pools that have a role in metabolism (Kauppinen et al., 1994; Rae, 2014; Stagg and Rothman, 2014). It has therefore been suggested that MRS-measured Glx and GABA+ should be interpreted as excitatory and inhibitory tone, rather than an exact measure of neuronal activity at the time of scanning (Farrant and Nusser, 2005; Harris et al., 2015; Rae, 2014). Having said this, tonic (in contrast to phasic) excitation and inhibition represented by ^1^H-MRS Glx and GABA+ are in fact highly relevant in our study, as not only do they represent activity in the resting state, in which young adult APOE E4 carriers have been shown to have hyperactivity and hyperconnectivity (Busche and Konnerth, 2016; Koelewijn et al., 2019), but also they predict both resting-state connectivity and task-related PMC (de)activation (Hu et al., 2013; Kapogiannis et al., 2013).

In addition, the spatial specificity of MRS (e.g. 2x2x3cm voxel) is much lower than fMRI, where a voxel is measured in millimetres. Any subtle changes in neurotransmitter levels arising in young E4 carriers may be diluted over the large voxel area. Indeed, there is evidence for the co-existence of functional unity but also diversity within the PMC (Parvizi et al., 2006; Yang et al., 2014). Large MRS voxel sizes are required to achieve a sufficient signal-to-noise for metabolites present at low concentrations. This is particularly challenging for GABA+, as its concentration referenced to water tends to be below 3 mM (Mullins et al., 2014; Rae, 2014). That said, ^1^H-MRS studies do show functionally relevant correlations between PMC Glx, GABA+ and E/I balance and DN deactivation (Gu et al., 2019; Hu et al., 2013), indicating ^1^H-MRS is sensitive to quantify the metabolites that relate to the BOLD signal. Moving to stronger magnetic fields, such as 7T, could improve sensitivity and reduce voxel size, through improving SNR and resolution of metabolites with overlapping signals (Pradhan et al., 2015, Terpstra et al., 2016)

### 4.1 Limitations

Unfortunately, several spectra were rejected due to not meeting our data quality criteria. This was particularly the case for PMC MEGA-PRESS spectra, which affected the sample sizes for GABA+ and Glx/GABA+. This was likely due to the smaller PMC voxel size, as fewer GABA+ spectra were rejected in the larger OCC voxel. The reduced voxel size was important to improve spatial specificity however, so increasing the PMC voxel size would not be a good way to address this. As discussed above, moving to 7T may reduce data exclusion as there is improved SNR at higher magnetic field strength. In future studies, when deciding on sample size we would recommend allowing for the exclusion of more MEGA-PRESS than PRESS spectra.

A reduction in sample size has an impact on power to detect our expected effects. To address this, a sensitivity analysis performed in G*Power reveals that updating the PMC sample sizes to 19 E4 carriers vs. 26 non-carriers for Glx, 14 vs 18 for GABA+ and 14 vs 17 for Glx/GABA+ would have 80% power to detect an effect at p<0.05 given an effect size of Cohen's d = 0.86, 1.03 or 1.05 respectively. These effect sizes are within the effect size of the previously detected PMC deactivation difference between APOE groups in a comparable population (Shine et al., 2015). Thus, despite reductions in sample size, we retained adequate power to detect a large effect between groups. Furthermore, our use of Bayes factors allowed us to assess the extent that our data provide evidence for or against the null hypothesis. Although replicable and precise results are more likely when statistical power is high (Button et al., 2013), it is entirely possible for even low-power experiments to have high evidential value, and contrastingly, for high-power experiments to have low evidential value (Dienes and Mclatchie, 2018). As discussed earlier, Bayes factors in the PMC voxel of interest showed that the data provide substantial evidence in favour of the null (Dienes, 2014).

An improvement to our study that would further inform whether the PM network hyperactivation seen in young APOE E4 carriers is related to shifted E/I balance would be to assess both fMRI and/or MEG and ^1^H-MRS within the same participants. Although reduced DN deactivation appears to be a consistent fMRI signature in APOE E4 carriers, correlating BOLD and metabolites in the same participants would provide more direct evidence of whether/how these factors are related. Moreover, a recent development in ^1^H-MRS literature which may reveal more subtle relationships between fMRI and ^1^H-MRS metabolites is functional ^1^H-MRS (fMRS), which could potentially detect task-related metabolite differences between APOE E4 carriers and non-carriers that are not possible to assess using conventional ^1^H-MRS collected at rest (Apšvalka et al., 2015; Huang et al., 2015; Mullins, 2018; Thielen et al., 2018). In addition, extending the age range of participants, or using a longitudinal design, would further enable us to track how DN deactivation, PMC metabolites, AD pathology and episodic cognition may change with age in APOE E4 carriers (Foster et al., 2018) to give greater insight into the timeline of how and when APOE E4 possession may predispose to development of AD.

### 4.2 Summary

This study provides evidence against differences in PMC Glx, GABA+ and E/I balance in young adult APOE E4 carriers versus non-carriers. This suggests that the hyperactivation (or reduced deactivation) previously observed in this region in APOE E4 carriers is unlikely to be directly associated with altered levels of local neurotransmitters, despite there being evidence from fMRI-^1^H-MRS studies that local Glx, GABA+ and E/I balance at rest are related to PMC task-related deactivation (Gu et al., 2019; Hu et al., 2013). Our null findings could inform models of the spatio-temporal ordering of alterations within the PMC of APOE E4 carriers that predispose to earlier amyloid accumulation in this region and ultimately development of AD, suggesting that resting PMC neurotransmitter differences do not occur prior to PMC functional changes that are observed in young adult APOE E4 carriers in this region. Alternatively, instead of local effects, it could suggest that PMC hyperactivation in APOE E4 carriers may be related to altered E/I balance or possibly other factors elsewhere in the PM network, a key candidate region being the MTL. Our study thus identifies areas for future investigation to gain better understanding of how and why activity differences in PMC occur in young APOE E4 carriers that subsequently predispose to earlier AD pathology.

## CRediT author statement

Costigan AG: Conceptualisation, Investigation, Formal analysis, Visualization, Writing- Original Draft; Umla-Runge, K: Conceptualisation, Investigation, Project administration; Evans, CJ: Methodology, Software, Validation; Raybould R: Investigation; Graham, KS: Conceptualisation, Project administration, Supervision; Lawrence, AD: Conceptualisation, Supervision, Writing – Review and Editing.

## Declaration of competing interest

The authors declare that they have no known competing financial interests or personal relationships that could have appeared to influence the work reported in this paper.
