# Supplementary material for: Evidence against altered excitatory/inhibitory balance in the posteromedial cortex of young adult APOE E4 carriers: A resting state 1H-MRS study
Source: Neuroimage Rep. 2021 Nov 11;1(4):100059. doi: 10.1016/j.ynirp.2021.100059 (PMC9986794; doi:10.1016/j.ynirp.2021.100059)
Supplement: Multimedia component 1 [file mmc1.docx]

Supplementary Material

# MRS pilot study: Test-retest reliability

## Introduction

The purpose of the pilot study was to assess the test-retest reliability of the six main ^1^H-MRS metabolites. In the PRESS scan these were Glx, N-acetyl-aspartic-acid (tNAA), myo-Inositol (mI), creatine (tCr) and choline (Cho), and GABA+ in the MEGA-PRESS scan.

## Methods

Six participants (3 male, 3 female, mean age 25.5 ± 1.6 years) took part in two scan sessions. The study was approved by the Cardiff University School of Psychology Research Ethics Committee, and written informed consent was obtained from all participants. As in the main text, scans were scheduled for the female participants in the luteal phase of the menstrual cycle, to avoid any effects of menstrual cycle phase on metabolite values. Scans were scheduled an average of 3 weeks apart.

Scans were performed at the Cardiff University Brain Research Imaging Centre (CUBRIC) on a 3T General Electric (GE) HDx scanner fitted with an 8-channel phased array head coil. The two scan sessions for each participant included a structural scan, two PMC PRESS scans, two OCC PRESS scans, and two PMC MEGA-PRESS scans. This meant that each scan type was repeated within a scan session, allowing for an intra-scan comparison of metabolite levels when the voxel was placed in the same position, and an inter-scan comparison between scan sessions. As in the main text, the PMC voxel measured 2x2x3cm, and the OCC voxel 3x3x3cm (see Figure 1). The same PRESS scan parameters as in section 2.4 were used, and the PMC MEGA-PRESS scans used the parameters TE = 68ms, TR = 1800ms, number of averages = 332, spectral width 5kHz, number of data points = 4096, acquisition time = 10minutes (i.e. a shorter scan time with fewer averages than in the main text). We did not measure GABA+ in OCC MEGA-PRESS scans, as a test-retest reliability assessment had been done previously in CUBRIC, and a 3x3x3cm OCC voxel was a standard method at this centre (NB. Our PMC MEGA-PRESS scan parameters matched that of the OCC voxel in these studies) (Mikkelsen et al., 2015; Muthukumaraswamy et al., 2009, 2012).

Data analysis was the same as described in section 2.5. Briefly, PRESS data (for Glx, tNAA, mI, TCr, Cho) were analysed using TARQUIN (Totally Automatic Robust Quantification In NMR) version 4.3.3 (Wilson et al., 2011). MEGA-PRESS data (for GABA+) were analysed using GANNET (GABA-MRS Analysis Tool) version 2.0 (Edden et al., 2014). Metabolite signals were referenced to H_2_O, corrected for the fraction of CSF in the voxel, and expressed as a concentration in millimoles (mM) per unit tissue volume. Data were excluded where the CRLB was above 20.

To test the intra-scan test-retest reliability, the coefficient of variation (CV) was calculated for the pairs of scans within each scan session (i.e. for scan 1a and 1b; and for scan 2a and 2b). For the inter-scan test-retest reliability, the coefficient of variation was calculated for the first scan in each session (i.e. for scan 1a and 2a). CV is calculated as (standard deviation/average x 100). Metabolite values from the four scans were also compared to assess if they were significantly different from each other, using a repeated measures ANOVA (SPSS).

## Results and Discussion

The following data were excluded due to CRLB>20: in the OCC voxel, two Cho, two Glx, three mI, two tCr and two tNAA values; in the PMC voxel, one mI value.

Supplementary table 1 lists the intra- and inter- scan CVs. There were no significant differences in any metabolite across the four scans (see Supplementary Table 1; all p>0.08).

Previous studies of PRESS scan test-retest reliability have reported CVs of 5% for tNAA, Cr and mI, and 9% for tCho in the cortex measured at 1.5T (Geurts et al., 2004), or 7% for tNAA, Cr and tCho, and 10% for mI at 3T (Kirov et al., 2012), and have suggested these values represent a reliable measurement. The CV for GABA+ quantified from a MEGA-PRESS scan acquired at 3T was 4% in the OCC and 14.8% in an anterior cingulate cortex voxel (OCC voxel measured 3x3x3cm, ACC voxel 2x3x4cm) (Mikkelsen et al., 2015). Our CVs are comparable to these findings.

The ability of Gannet to fit the GABA+ data to the model was noted to be lower for the PMC GABA+ data than found for OCC GABA+ data in CUBRIC, indicated by larger residuals in the PMC data. Therefore, to improve PMC GABA+ data quality in our main study, the scan time was lengthened from 10 to 15 minutes to acquire an extra 180 averages.

| **MRS voxel** | **Metabolite** | **Data exclusions (CRLB>20)** | **Intra-scan CV for scans 1a and 1b** | **Intra-scan CV for scans 2a and 2b** | **Average intra-scan CV** | **Inter-scan CV for scans 1a and 2a** | **Repeated measures ANOVA across the four scans** |
| --- | --- | --- | --- | --- | --- | --- | --- |
|  |  |  |  |  |  |  |  |
| OCC | Cho | 2 | 2.69 | 4.39 | 3.54 | 8.15 | F(3,9)=2.54, p=0.12 |
|  | Glx | 2 | 12.86 | 13.98 | 13.42 | 12.88 | F(3,9)=0.19, p=0.90 |
|  | mI | 3 | 2.30 | 8.60 | 5.45 | 9.35 | F(3,6)=0.81, p=0.53 |
|  | tCr | 2 | 2.74 | 5.64 | 4.19 | 7.56 | F(3,9)=1.24, p=0.35 |
|  | tNAA | 2 | 4.07 | 7.03 | 5.55 | 5.04 | F(3,9)=2.07, p=0.25 |
|  |  |  |  |  |  |  |  |
|  |  |  |  |  |  |  |  |
| PMC | Cho |  | 2.76 | 5.23 | 3.99 | 5.26 | F(3,15)=2.81, p=0.08 |
|  | Glx |  | 7.43 | 10.31 | 8.87 | 11.97 | F(3,15)=0.86, p=0.49 |
|  | mI | 1 | 5.18 | 5.29 | 5.24 | 10.38 | F(3,12)=0.36, p=0.79 |
|  | tCr |  | 2.75 | 3.04 | 2.90 | 6.07 | F(3,15)=1.91, p=0.17 |
|  | tNAA |  | 5.42 | 4.28 | 4.85 | 3.71 | F(3,15)=1.99, p=0.16 |
|  | GABA+ |  | 16.77 | 10.05 | 13.41 | 12.61 | F(3,15)=2.37, p=0.11 |
|  |  |  |  |  |  |  |  |

**Supplementary Table 1**: Intra- and inter-scan coefficient of variation (CV) and statistical analysis across four scans to assess test-retest reliability of PRESS metabolites in the OCC and PMC voxels, and of MEGA-PRESS GABA+ in the PMC voxel.

# Comparison of other main ^1^H-MRS metabolites: replication of Suri et al. (2017)

## Introduction

The ^1^H-MRS scan sequence with a TE of 35ms used to quantify Glx in the main text also allows for quantification of a range of metabolites commonly studied in ^1^H-MRS papers. The main metabolites of interest, which are the most often studied and of greatest relevance to APOE E4 carriers as they are altered in AD, are N-acetyl-aspartic-acid (tNAA), myo-Inositol (mI), creatine (tCr) and choline (Cho).

NAA is interpreted as a marker of neuronal density and integrity, as it is synthesized predominantly in neuronal mitochondria (Patel & Clark, 1979; Rae, 2014). It is considered a neuronal marker as tNAA is negatively correlated with atrophy in neurodegenerative disorders (Oz et al., 2010, 2014), and positively correlated with synapse density in a 1H-MRS study of elderly participants followed by histology on post-mortem tissue (Murray et al., 2014). The “t” in tNAA denotes “total”, as the large NAA peak in the MRS spectrum has a small peak on its shoulder from N-acetyl-aspartic glutamate (NAAG), which has a highly similar chemical structure, so is difficult to accurately separate from NAA (Edden et al., 2007). Therefore, these two signals are combined and reported together as standard.

MI is considered to be a marker of inflammation. It is associated with gliosis, the recruitment and proliferation of glial cells to an affected region as a response to injury or disease (Rae, 2014). This was established first in cancer (glioma) and subsequently in other neuroinflammatory disorders such as multiple sclerosis, as well as acute inflammation due to traumatic brain injury (Oz et al., 2014).

Similar to tNAA, tCr is the sum of the peaks on the MRS spectrum from Cr and phosphocreatine (PCr), which is the product of the phosphorylation of Cr by creatine kinase (Rae, 2014). This reaction is termed the creatine kinase/phosphocreatine (CK/PCr) energy shuttle, and is an important way that the cell replenishes its supply of ATP. TCr, therefore, is used as a marker of energy metabolism.

The Cho signal represents several choline containing compounds, which include free choline (Cho), phosphocholine (PC) and glycerophosphocholine (GPC) (Barker et al., 1994; Miller et al., 1996). Membrane-bound choline in phospholipids is MRS-invisible (Miller et al., 1991), thus the signal obtained in MRS represents cytosolic choline compounds (Stagg & Rothman, 2014). In MRS, the Cho peak is considered a marker of cell membrane integrity or turnover (Rae, 2014).

In ^1^H-MRS studies of AD patients, a decrease in PMC tNAA and an increase in PMC mI is a robust finding replicated several times (Kantarci et al., 2000, 2007, 2013; Murray et al., 2014; Voevodskaya et al., 2016; H. Wang et al., 2015). The increase in PMC mI may precede the decrease in tNAA, as one study found that increased mI was detected in both AD and MCI patients, while decreased PMC tNAA was only detected in AD patients (Kantarci et al., 2000). This suggests AD is characterized by an increase in inflammation and a decrease in neuronal density in this brain region. Changes in Cho are less consistent, but there are reports of an elevation in Cho (Kantarci et al., 2004, 2007). Many studies use Cr as a reference for other metabolites, so tCr tends not to be reported alone, however this is cautioned against as any tCr changes in AD could lead to misleading results for the other metabolites (Rae, 2014; Stagg & Rothman, 2014).

Some studies have detected ^1^H-MRS metabolite changes in older APOE E4 carriers in the PMC. Differences include higher mI in carriers compared to non-carriers (Voevodskaya & Sundgren, 2016), higher mI/Cr and Cho/Cr (Gomar et al., 2014), lower Cr (Laakso et al., 2003) and lower tNAA (Riese et al., 2015). This pattern of metabolite differences between older E4 carriers and non-carriers have not always been detected however (Kantarci et al., 2002; Laakso et al., 2003; Suri et al., 2017).

A key study of note here is by Suri et al. (2017) which analysed PMC metabolites in a group of old (age 60-85 years) and young participants (age 20-40), which was also split into APOE E4 carriers and non-carriers. They studied a 2x2x2cm PMC voxel, placed in a highly similar location to our PMC voxel. They found a significant effect of age on certain metabolites, where older participants had higher PMC mI and tCr, and lower PMC glutamate. However, there was no significant effect of APOE group or interaction of APOE group and age on any metabolite. Although this paper had a large sample size in their older group (n=117 participants, inc. 22 E4 carriers), a limitation of the study is that their younger group only had a sample size of 8 APOE E4 carriers compared to 22 non-carriers. This could limit the power to detect an effect, as it is lower than the sample size often used in MRS studies people at genetic risk of disease with controls (e.g. 22 participants at genetic risk of schizophrenia vs 22 controls (Yoo et al., 2009) and 23 risk participants vs 24 controls (Tandon et al., 2013)).

The purpose of our analysis was to use existing data from our Glx analysis to replicate Suri et al. (2017), testing whether young APOE E4 carriers have any differences in the four main metabolites studied in AD compared to non-carriers (tNAA, mI, tCr, Cho). Advantages of our study are that we had a larger sample size of young E4 carriers (n=20 carriers vs 27 non-carriers), and we included an OCC voxel as a control to test whether any metabolite differences were region specific.

## Methods

The methods are the same as in the main text. The four metabolites analyzed here were quantified in the same analysis of the PRESS scan data for Glx using Tarquin v4.3.3, but not included in the main text as our focus there was on Glx and GABA.

Data excluded due to CRLBs over 20 were, in the PMC, two tNAA (two E4 carriers), 14 mI (four E4 carriers, 10 non-carriers), two tCr (one E4 carrier, one non-carrier), three Cho (one E4 carrier, two non-carriers), and in the OCC, one tNAA, four mI, one tCr and two Cho (all E4 carriers).

## Results and Discussion

There were no differences in the concentrations of the four metabolites between APOE E4 carriers in and non-carriers in the PMC voxel of interest: tNAA, t(42)=0.85, p=0.40, Cohen’s d = -0.26, BF_01_=2.49; mI, t(31)=0.70, p=0.49, Cohen’s d= -0.24, BF_01_=2.49; tCr, t(43)=0.03, p=0.97, Cohen’s d= -0.01, BF_01_=3.36; tCho, t(42)=0.62, p=0.54, Cohen’s d= -0.19, BF_01_=2.86.

There were also no differences between E4 carriers and non-carriers in the OCC comparison voxel: tNAA, t(44)=1.09, p=0.28, Cohen’s d = -0.33, BF_01_=2.09; mI, t(41)=0.0005, p=1.00, Cohen’s d= 0.0001, BF_01_=3.24; tCr, t(44)=0.93, p=0.36, Cohen’s d= -0.28, BF_01_=2.39; tCho, t(43)=0.97, p=0.34, Cohen’s d= -0.29, BF_01_=2.30.

Our PMC findings therefore replicate the null findings of Suri et al. (2017) in the younger APOE E4 carriers and suggest that findings in older adults may reflect consequences of aging/disease related processes.

**Supplementary Figure 1:** Comparison of ^1^H-MRS metabolites in APOE E4 carriers and non-carriers in the PMC voxel of interest and OCC comparison voxel. Plots show individual data points, lines represent the mean, and error bars are 95% confidence intervals.

**Acknowledgements**

We are grateful to Mark Mikkelsen for sharing Matlab scripts for ^1^H-MRS analysis, and to Peter Hobden for assistance with scanning. The work was funded by the Wellcome Trust (Strategic Award – KSG, ADL, 104943/Z/14/Z) and Medical Research Council (KSG, G1002149). AGC’s PhD studentship was funded by the Cardiff University Neuroscience and Mental Health Research Institute.

**CRediT author statement**

**Costigan AG:** Conceptualisation, Investigation, Formal analysis, Visualization, Writing- Original Draft; **Umla-Runge, K:** Conceptualisation, Investigation, Project administration; **Evans, CJ:** Methodology, Software, Validation; **Raybould R:** Investigation; **Graham, KS:** Conceptualization, Project administration, Supervision; **Lawrence, AD:** Conceptualisation, Supervision, Writing – Review and Editing.

**Bibliography**

Barker, P.B., Breiter, S.N., Soher, B.J., Chatham, J.C., Forder, J.R., Samphilipo, M.A., Magee C.A., Andreson, J.H. (1994). Quantitative proton spectroscopy of canine brain: in vivo and in vitro correlations. *Magnetic Resonance inMedicine, 32(2), 157-163*

Edden, R. A. E., Pomper, M. G., & Barker, P. B. (2007). *In Vivo Differentiation of N-Acetyl Aspartyl Glutamate From N-Acetyl Aspartate at 3 Tesla*. *982*(December 2006), 977–982. https://doi.org/10.1002/mrm.21234

Edden, R. A. E., Puts, N. A. J., Harris, A. D., Barker, P. B., & Evans, C. J. (2014). Gannet: A batch-processing tool for the quantitative analysis of gamma-aminobutyric acid-edited MR spectroscopy spectra. *Journal of Magnetic Resonance Imaging*, *40*(6), 1445–1452. https://doi.org/10.1002/jmri.24478

Geurts, J. J. G., Barkhof, F., Castelijns, J. a, Uitdehaag, B. M. J., Polman, C. H., & Pouwels, P. J. W. (2004). Quantitative 1H-MRS of healthy human cortex, hippocampus, and thalamus: metabolite concentrations, quantification precision, and reproducibility. *Journal of Magnetic Resonance Imaging : JMRI*, *20*(3), 366–371. https://doi.org/10.1002/jmri.20138

Gomar, J. J., Gordon, M. L., Dickinson, D., Kingsley, P. B., Uluğ, A. M., Keehlisen, L., Huet, S., Buthorn, J. J., Koppel, J., Christen, E., Conejero-Goldberg, C., Davies, P., & Goldberg, T. E. (2014). APOE Genotype Modulates Proton Magnetic Resonance Spectroscopy Metabolites in the Aging Brain. *Biological Psychiatry*, *75*(9), 686–692. https://doi.org/10.1016/j.biopsych.2013.05.022

Kantarci, K., Jack Jr, C. R., Xu, T. C., Campeau, N. G., O’Brien, P. C., Smith, G. E., Ivnik, R. J., Boeve, B. F., Kokmen, E., Tangalos, E. G., & Petersen, R. C. (2000). Regional metabolic patterns in mild cognitive impairment and Alzheimer’s disease: A 1H-MRS Study. *Neurology*, *55*(2), 210–217.

Kantarci, K., Petersen, R. C., Boeve, B. F., David, S., Tang-wai, D. F., OBrien, P. C. O., Weigand, S. D., Edland, S. D., Smith, G. E., Ivnik, R. J., Ferman, T. J., Tangalos, E. G., & Jr, C. R. J. (2004). 1H MR Spectroscopy in Common Dementias. *Neurology*, *63*(8), 1393–1398. https://doi.org/https://doi.org/10.1212/01.WNL.0000141849.21256.AC

Kantarci, K., Smith, G. E., Ivnik, R. J., Petersen, R. C., Bradley, F., Knopman, D. S., Tangalos, E. G., & Jr, C. R. J. (2002). 1H Magnetic Resonance Spectroscopy, Cognitive Function, and Apolipoprotein E Genotype in Normal Aging, Mild Cognitive Impairment and Alzheimer’s Disease. *J Int Neuropsychol Soc*, *8*(7), 934–942.

Kantarci, K., Weigand, S. D., Petersen, R. C., Boeve, B. F., Knopman, D. S., Gunter, J., Reyes, D., Shiung, M., O’Brien, P. C., Smith, G. E., Ivnik, R. J., Tangalos, E. G., & Jack, C. R. (2007). Longitudinal 1H MRS changes in mild cognitive impairment and Alzheimer’s disease. *Neurobiology of Aging*, *28*(9), 1330–1339. https://doi.org/10.1016/j.neurobiolaging.2006.06.018

Kantarci, K., Weigand, S. D., Przybelski, S. A., Preboske, G. M., Pankratz, V. S., Murphy, M. C., Gunter, J. L., Machulda, M. M., Ivnik, R. J., Roberts, R. O., Rocca, W. A., Knopman, D. S., & Petersen, R. C. (2013). MRI and MRS predictors of mild cognitive impairment in a population-based sample. *Neurology*, *81*, 126–133.

Kirov, I. I., George, I. C., Jayawickrama, N., Babb, J. S., Perry, N. N., & Gonen, O. (2012). Longitudinal inter- and intra-individual human brain metabolic quantification over 3 years with proton MR spectroscopy at 3 T. *Magnetic Resonance in Medicine : Official Journal of the Society of Magnetic Resonance in Medicine / Society of Magnetic Resonance in Medicine*, *67*(1), 27–33. https://doi.org/10.1002/mrm.23001

Laakso, M. P., Hiltunen, Y., Könönen, M., Kivipelto, M., Koivisto, A., Hallikainen, M., & Soininen, H. (2003). Decreased brain creatine levels in elderly apolipoprotein E epsilon 4 carriers. *Journal of Neural Transmission (Vienna, Austria : 1996)*, *110*(3), 267–275. https://doi.org/10.1007/s00702-002-0783-7

Mikkelsen, M., Singh, K. D., Sumner, P., & Evans, C. J. (2015). Comparison of the Repeatability of GABA-Edited Magnetic Resonance Spectroscopy with and without Macromolecule Suppression. *Magnetic Resonance in Medicine*, *00*, 1–8. https://doi.org/10.1002/mrm.25699

Miller BL, Chang L, Booth R, Ernst T, Cornford M, Nikas D, McBride D, Jenden DJ. In vivo 1H MRS choline: correlation with in vitro chemistry/histology. Life Sci. 1996;58(22):1929-35.

Murray, M. E., Przybelski, S. a., Lesnick, T. G., Liesinger, a. M., Spychalla, a., Zhang, B., Gunter, J. L., Parisi, J. E., Boeve, B. F., Knopman, D. S., Petersen, R. C., Jack, C. R., Dickson, D. W., & Kantarci, K. (2014). Early Alzheimer’s Disease Neuropathology Detected by Proton MR Spectroscopy. *Journal of Neuroscience*, *34*(49), 16247–16255. https://doi.org/10.1523/JNEUROSCI.2027-14.2014

Muthukumaraswamy, S. D., Edden, R. A. E., Jones, D. K., Swettenham, J. B., & Singh, K. D. (2009). Resting GABA concentration predicts peak gamma frequency and fMRI amplitude in response to visual stimulation in humans. *PNAS*, *106*(20), 2–7. https://doi.org/https://doi.org/10.1073/pnas.0900728106

Muthukumaraswamy, S. D., Evans, C. J., Edden, R. a E., Wise, R. G., & Singh, K. D. (2012). Individual variability in the shape and amplitude of the BOLD-HRF correlates with endogenous GABAergic inhibition. *Human Brain Mapping*, *33*(2), 455–465. https://doi.org/10.1002/hbm.21223

Oz, G., Alger, J. R., Barker, P. B., Bartha, R., Bolan, P. J., Brindle, K. M., Cudalbu, C., Howe, F. A., Hüppi, P. S., & Hurd, R. E. (2014). Clinical Proton MR Spectroscopy in Central Nervous System. *Radiology*, *270*(3).

Oz, G., Nelson, C. D., Koski, D. M., Henry, P.-G., Marjanska, M., Deelchand, D. K., Shanley, R., Eberly, L. E., Orr, H. T., & Clark, H. B. (2010). Noninvasive detection of presymptomatic and progressive neurodegeneration in a mouse model of spinocerebellar ataxia type 1. *The Journal of Neuroscience : The Official Journal of the Society for Neuroscience*, *30*(10), 3831–3838. https://doi.org/10.1523/JNEUROSCI.5612-09.2010

Patel, T. B., & Clark, J. B. (1979). Synthesis of N-acetyl-l-aspartate by rat brain mitochondria and its involvement in mitochondrial/cytosolic carbon transport. *Biochemical Journal*, *184*(3), 539–546.

Rae, C. D. (2014). A Guide to the Metabolic Pathways and Function of Metabolites Observed in Human Brain 1H Magnetic Resonance Spectra. *Neurochemical Research*, *39*(1), 1–36. https://doi.org/10.1007/s11064-013-1199-5

Riese, F., Gietl, A., Zölch, N., Henning, A., Gorman, R. O., Kälin, A. M., Leh, S. E., Buck, A., Warnock, G., Edden, R. A. E., Luechinger, R., Hock, C., Kollias, S., & Michels, L. (2015). Posterior cingulate gamma-aminobutyric acid and glutamate / glutamine are reduced in amnestic mild cognitive impairment and are unrelated to amyloid deposition and apolipoprotein E genotype. *Neurobiology of Aging*, *36*(1), 53–59. https://doi.org/10.1016/j.neurobiolaging.2014.07.030

Stagg, C. J., & Rothman, D. L. (Eds.). (2014). *Magnetic Resonance Spectroscopy: Tools for Neurosceicne Research and Emerging Clinical Applications*. Elsevier.

Suri, S., Mackay, C. E., Kelly, M. E., Germuska, M., Tunbridge, E. M., Frisoni, G. V. B., Matthews, P. M., Ebmeier, K. P., Bulte, D. P., & Filippini, N. (2015). Reduced cerebrovascular reactivity in young adults carrying the APOE ε4 allele. *Alzheimer’s and Dementia*, *11*(6), 648–657. https://doi.org/10.1016/j.jalz.2014.05.1755

Tandon, N., Bolo, N. R., Sanghavi, K., Mathew, I. T., Francis, A. N., Stanley, J. a, & Keshavan, M. S. (2013). Brain metabolite alterations in young adults at familial high risk for schizophrenia using proton magnetic resonance spectroscopy. *Schizophrenia Research*, *148*(1–3), 59–66. https://doi.org/10.1016/j.schres.2013.05.024

Voevodskaya, O., Sundgren, P. C., Strandberg, O., Zetterberg, H., Minthon, L., Blennow, K., Wahlund, L.-O., Westman, E., & Hansson, O. (2016). Myo-inositol changes precede amyloid pathology and relate to APOE genotype in Alzheimer disease. *Neurology*, *86*, 1–8.

Wang, H., Tan, L., Wang, H.-F., Liu, Y., Yin, R.-H., Wang, W.-Y., Chang, X.-L., Jiang, T., & Yu, J.-T. (2015). Magnetic Resonance Spectroscopy in Alzheimer’s Disease: Systematic Review and Meta-Analysis. *Journal of Alzheimer’s Disease*, *46*(4), 1049–1070. https://doi.org/10.3233/JAD-143225

Wilson, M., Reynolds, G., Kauppinen, R. a, Arvanitis, T. N., & Peet, A. C. (2011). A constrained least-squares approach to the automated quantitation of in vivo 1 H magnetic resonance spectroscopy data. *Magnetic Resonance in Medicine*, *65*(1), 1–12. https://doi.org/10.1002/mrm.22579

Yoo, S. Y., Yeon, S., Choi, C.-H., Kang, D.-H., Lee, J.-M., Shin, N. Y., Jung, W. H., Choi, J.-S., Jang, D.-P., & Kwon, J. S. (2009). Proton magnetic resonance spectroscopy in subjects with high genetic risk of schizophrenia: investigation of anterior cingulate, dorsolateral prefrontal cortex and thalamus. *Schizophrenia Research*, *111*(1–3), 86–93. https://doi.org/10.1016/j.schres.2009.03.036
